# Vascular and Tissue Changes of Magnetic Susceptibility in the Mouse Brain After Transient Cerebral Ischemia

**DOI:** 10.1007/s12975-017-0591-x

**Published:** 2017-11-25

**Authors:** Markus Vaas, Andreas Deistung, Jürgen R. Reichenbach, Annika Keller, Anja Kipar, Jan Klohs

**Affiliations:** 1 Institute for Biomedical Engineering, University of Zurich and ETH Zurich, Vladimir-Prelog-Weg 4, 8093 Zurich, Switzerland; 2 Neuroscience Center Zurich, University of Zurich and ETH Zurich, Zurich, Switzerland; 3 Medical Physics Group, Institute of Diagnostic and Interventional Radiology, University Hospital Jena, 07743 Jena, Germany; 4 Section of Experimental Neurology, Department of Neurology, Essen University Hospital, 45147 Essen, Germany; 5 Erwin L. Hahn Institute for Magnetic Resonance Imaging, University Duisburg-Essen, 45141 Essen, Germany; 6 Michael Stifel Center for Data-driven and Simulation Science Jena, Friedrich Schiller University Jena, 07743 Jena, Germany; 7 Division of Neurosurgery, University Hospital Zurich, 8091 Zurich, Switzerland; 8 Institute of Veterinary Pathology, University of Zurich, 8057 Zurich, Switzerland

**Keywords:** Quantitative susceptibility mapping, MR frequency, Magnetic resonance imaging, Mice, Middle cerebral artery occlusion, Ischemia

## Abstract

Quantitative susceptibility mapping (QSM) has been recently introduced as a novel MRI post-processing technique of gradient recalled echo (GRE) data. QSM is useful in depicting both brain anatomy and for detecting abnormalities. Its utility in the context of ischemic stroke has, however, not been extensively characterized so far. In this study, we explored the potential of QSM to characterize vascular and tissue changes in the transient middle cerebral artery occlusion (tMCAO) mouse model of cerebral ischemia. We acquired GRE data of mice brains at different time points after tMCAO, from which we computed QSM and MR frequency maps, and compared these maps with diffusion imaging and multi-slice multi-echo imaging data acquired in the same animals. Prominent vessels with increased magnetic susceptibility were visible surrounding the lesion on both frequency and magnetic susceptibility maps at all time points (mostly visible at > 12 h after reperfusion). Immunohistochemistry revealed the presence of compressed capillaries and dilated larger vessels, suggesting that the appearance of prominent vessels after reestablishment of reperfusion may serve compensatory purposes. In addition, on both contrast maps, tissue regions of decreased magnetic susceptibility were observed at 24 and 48 h after reperfusion that were distinctly different from the lesions seen on maps of the apparent diffusion coefficient and *T*_2_ relaxation time constant. Since QSM can be extracted as an add-on from GRE data and thus requires no additional acquisition time in the course of acute stroke MRI examination, it may provide unique and complementary information during the course of acute stroke MRI examinations.

## Introduction

Magnetic resonance imaging (MRI) is an important aid for physicians in the diagnosis and management of patients with acute stroke [1], providing multiple useful contrasts for assessing hemodynamic function as well as extent and severity of brain injury. In case of ischemic stroke, magnetic resonance angiography, for instance, can identify occlusion of a parent artery [2], whereas perfusion-weighted imaging (PWI) informs about regional disturbances of cerebral blood supply in hyperacute and acute ischemic stroke [3]. Diffusion-weighted imaging (DWI) has been shown to depict the ischemic lesion in the hyperacute, acute, and subacute stage after an ischemic insult [4–7]. Analyzing *T*_1_ and *T*_2_ relaxation times has also been used to assess ischemic damage [8, 9].

Bulk magnetic susceptibility is a fundamental physical property representing a materials’ tendency to interact with and distort an applied magnetic field. By applying gradient (recalled) echo (GRE) magnetic resonance-based techniques, such as *T*_2_*-weighted imaging [10, 11], phase imaging [12, 13], and susceptibility-weighted imaging (SWI) [14, 15], it is possible to assess qualitatively magnetic susceptibility variations in the brain. Regarding acute stroke MRI, *T*_2_*-weighted imaging and SWI are used to detect cerebral microbleeds and hemorrhages [16], where SWI is also used to identify areas of hypoperfusion and to detect acute intravascular emboli [1]. Furthermore, asymmetrical veins between ischemic and normal brain tissues have been demonstrated with SWI, which may add information about local oxygen metabolism [17–19].

More recently, quantitative susceptibility mapping (QSM) has been introduced as a promising post-processing technique based on GRE data. QSM utilizes the small magnetic field variations arising from the underlying tissue magnetic susceptibility distribution to compute quantitative maps. It provides complementary anatomical contrast of the brain [20, 21], supports identification and characterization of brain lesions [22, 23], but also enables quantification of tissue iron content [24, 25], assessment of functional changes [26], and quantification of contrast agent concentration [27]. Concerning acute stroke QSM has been shown to be able to assess vessel function and oxygen metabolism in patients with acute stroke [18, 28] as well as in animal models of the disease [29].

In the present study, we investigated the potential of QSM and MR frequency mapping to assess the evolution of vascular and tissue changes in the mouse brain after transient middle cerebral artery occlusion (tMCAO). We acquired high-resolution GRE, DWI, and multi-slice multi-echo imaging data of mice brains at different time points after reperfusion. On the post-processed QSM and MR frequency maps, magnetic susceptibility and frequency were quantified in prominent vessels and brain tissues in both the ischemic and contralateral hemisphere side. We also evaluated the time courses of the occurrence of regional contrast changes on the frequency, magnetic susceptibility, apparent diffusion coefficient (ADC), and *T*_2_ relaxation time constant maps. Immunohistochemical analyses were performed to assess underlying vascular pathology.

## Methods

### Animals

All procedures conformed to the national guidelines of the Swiss Federal act on animal protection and were approved by the Cantonal Veterinary Office Zurich (Permit Number: 18-2014 and 49-2011). All procedures fulfilled the ARRIVE guidelines on reporting animal experiments. Animals were housed in a temperature-controlled room in individually ventilated cages, containing up to five animals per cage, under a 12-h dark/light cycle. Paper tissue was given as environmental enrichment. Access to pelleted food (3437PXL15, CARGILL) and water was provided ad libitum.

### Study Design and Ischemia Model

Seventeen male C57Bl6/J mice (Janvier, France, weight range 20–25 g, age range 8–10 weeks) were used. Anesthesia was initiated by using 3% isoflurane (Abbott, Cham, Switzerland) in a mixture of O_2_ (200 ml/min) and air (800 ml/min) and maintained with 1.5–2% isoflurane. Prior to surgery, a local analgesic (lidocaine, 0.5%, 7 mg/kg) was administered subcutaneously. Temperature was controlled during the surgery and kept constant at 36.5 ± 0.5 °C with a feedback-controlled heating pad system. The surgical procedure was carried out as described [30, 31]. The middle cerebral artery was occluded for 1 h. After surgery, buprenorphine was administered as subcutaneous injection every 6–8 h on the day of surgery (Temgesic, 0.1 mg/kg b.w) and supplied thereafter via the drinking water (1 mg/kg) for 36 h.

tMCAO animals were assessed with MRI at 2 h (*n* = 3), 4 h (*n* = 4), 6 h (*n* = 3), 12 h (*n* = 4), 24 h (*n* = 3), and 48 h (*n* = 6) after reperfusion, with the majority of animals being measured at two time points. ADC maps of all investigated mice were inspected. Animals were analyzed when a lesion was present on the ADC maps.

### Magnetic Resonance Imaging

MRI measurements were acquired on a Bruker PharmaScan 47/16 (Bruker BioSpin GmbH, Ettlingen, Germany) operating at 4.7 T and equipped with a cryogenic transmit-receive RF coil [32]. During MRI, mice were spontaneously breathing under isoflurane anesthesia (1.5%). Body temperature was monitored with a rectal temperature probe (MLT415, ADInstruments, Spechbach, Germany) and kept at 36 ± 0.5 °C using a warm water circuit integrated into the animal support.

Anatomical reference data acquired in coronal and sagittal orientations served for accurate positioning of the animal’s head. Global first-order shimming followed by fieldmap-based local shimming was performed on the mouse brain using the automated MAPshim routine to reduce field inhomogeneities.

For DWI, a two-dimensional (2D) multi-segment spin echo sequence with echo planar imaging readout (SE-EPI) was used. The scan parameters were field-of-view (FOV) = 17 mm × 14 mm, acquisition matrix = 128 × 128, nominal in-plane voxel size = 133 μm × 109 μm, 12 slices of 1 mm thickness, and an interslice distance = 1.3 mm, number of segments = 4, echo time (TE) = 27.5 ms, and repetition time (TR) = 3000 ms. Diffusion-encoding was applied in *x*-, *y*-, and *z*-direction (gradient pulse duration = 4 ms, gradient pulse separation = 14 ms) with *b* values of 100, 200, 400, 600, 800, and 1000 s/mm^2^, respectively. The acquisition time was 3 min and 48 s.

To extract *T*_2_ relaxation time constant of the brain tissue, a 2D Carr-Purcell-Meiboom-Gill multi-slice multi-echo sequence was applied with FOV = 20 mm × 20 mm, acquisition matrix = 100 × 100, nominal in-plane voxel size = 200 μm × 200 μm, 14 echoes with TE_1_ = 12 ms and an inter-echo time = 12 ms, TR = 2783 ms, and four averages. The acquisition time was 14 min and 6 s.

For frequency mapping and QSM, a 3D multi-echo GRE sequence was applied using a FOV = 25.6 mm × 25.6 mm × 8 mm and an acquisition matrix = 256 × 256 × 80, resulting in an effectively isotropic spatial resolution of 100 μm × 100 μm × 100 μm. Four echoes were recorded (TE_1–4_ = 4.5/10.5/16.5/22.5 ms) with TR = 100 ms, flip angle = 15°, monopolar echo readout and no averaging. The acquisition time was 25 min and 36 s.

### Data Processing

ADC maps were calculated on a pixel-by-pixel basis with linear regression analysis using the model function:

1 $$ \ln \left(S(b)/{S}_0\right)=-b\cdot \mathrm{ADC}, $$

where *S*(*b*) is the measured signal intensity at a specific *b* value (*b*) and *S*_0_ the signal intensity in the absence of a diffusion gradient (*b* = 0).

The T_2_ relaxation time was computed by fitting the spin echo magnitude signal, *S*, at each TE, to a mono-exponential decay function for each pixel using Paravision software (Bruker):

2 $$ S\left(\mathrm{TE}\right)={S}_0\cdot \exp \left(-\frac{\mathrm{TE}}{T_2}\right), $$

where both *S*_0_, the signal at TE = 0, and T_2_, the irreversible transverse relaxation time, are fit parameters.

Single-channel GRE magnitude images were combined using the sum-of-squares method [33], whereas single-channel GRE phase images were combined by taking the argument of the complex summed single-channel images after subtracting the channel-dependent phase offset estimated in the center of the 3D volume of the first echo [34]. Quantitative susceptibility maps were computed based on these combined phase images. To this end, the combined phase images for each echo were unwrapped using a 3D best-path algorithm [35], divided by (2*π* · TE) to obtain the Larmor frequency variation in Hz, and then combined across the different TEs in an optimized way that takes into account the local echo time-dependent contrast-to-noise ratio of the Larmor frequency images [36]. Background frequency contributions were eliminated using sophisticated harmonic artifact removal for phase data (SHARP) [37], with ten different spherical kernels with varying radii ranging from 100 to 1000 μm [38], and employing a regularization parameter for truncated singular value decomposition of 0.05. Susceptibility mapping was performed based on SHARP-processed frequency images using homogeneity enabled incremental dipole inversion (HEIDI) [20].

### Volume-of-Interest Analysis

Significant deviations from the signal distribution were identified in the ischemic hemisphere compared to the unaffected, contralateral hemisphere on all contrasts using Paravision (Bruker) and MRIcron. For quantitative evaluation, volumes-of-interest (VOIs) were drawn around three individual vessel structures that appeared prominent on both the ipsilateral side and the contralateral side if visible, and around one lateral ventricle using MRIcron (www.sph.sc.edu/comd/rorden/mricron). In addition, VOIs were drawn around areas of regional contrast change, as well as the remaining ipsi- and contralateral striatum and cortex. The extent of regional contrast changes was calculated edema corrected as described [39].

### Immunohistochemistry

After MRI, few mice were used for immunohistochemistry: 12 h (*n* = 2), 24 h (*n* = 3), and 48 h (*n* = 2) after reperfusion. Animals were deeply anesthetized by intraperitoneal injection of ketamine/xylazine/acepromazine maleate (100/20/3 mg/kg body weight) and decapitated. Brains were immediately removed and snap-frozen in 2-methylbutane (Sigma-Aldrich, Switzerland) cooled with dry ice to − 30 °C and stored at − 80 °C until processing. They were then thawed and fixed in 4% paraformaldehyde (PFA) for 48 h, then trimmed (coronal section) and embedded routinely in paraffin wax. Consecutive sections (3–5 μm) were prepared and, after antigen retrieval via incubation in citrate buffer (pH 6.0) for 20 min at 98 °C, incubated, with rabbit anti-mouse collagen IV (Cat # 2150-1470, AbD Serotec, dil 1:200) for 15–18 h at 4 °C. Subsequently, they were incubated with Envision rabbit, Dako.

### Statistical Analysis

Statistical analysis was performed using SigmaPlot 12.5 (Systat Software, San Jose, CA). Frequency values and susceptibility differences of vessels were compared with a Mann-Whitney rank sum test, whereas comparisons between different brain regions were performed using an analysis of variance, followed by Holm-Sidak post hoc test for multiple comparisons. Lesion volumes between different contrasts were compared with Student’s *t* test.

## Results

All post-processed images are made available in a data repository (10.6084/m9.figshare.5630071.v1).

### Prominent Vessels Within the Ischemic Hemisphere on Frequency and Susceptibility Maps

**Fig. 1:**
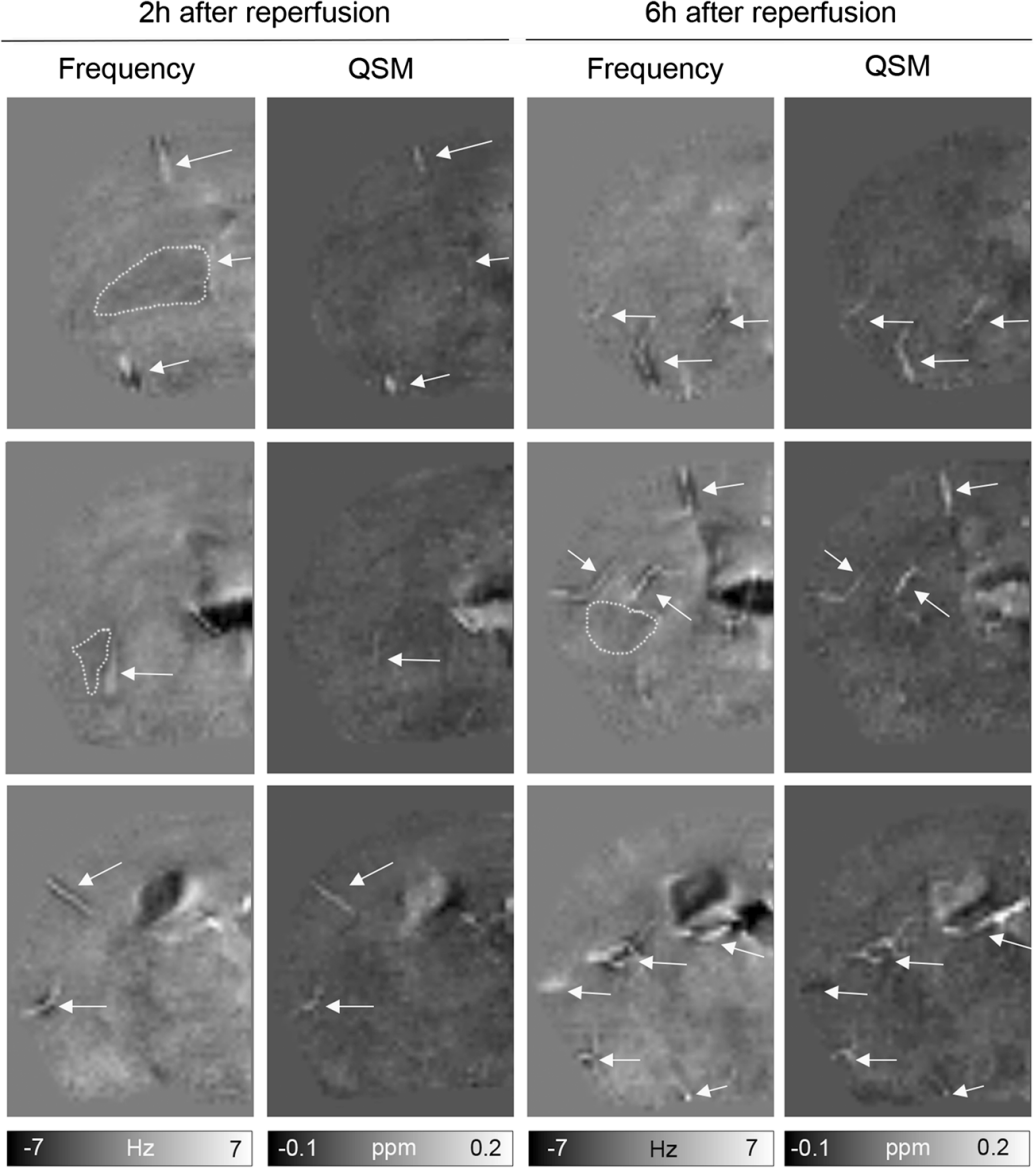
Display of representative axial background-field corrected frequency and quantitative susceptibility maps (QSM) of the ischemic hemisphere of a mouse of a tMCAO mouse after 2 and 6 h of reperfusion. For both contrasts, three cross-sections containing the ischemic territory (approximately bregma 0.14 and − 0.82 mm) are shown. Only few prominent vessels are seen with high MR frequencies and increased magnetic susceptibilities (white arrows). Lesions showing decreased frequencies are also discernable (enclosed by white dotted line)

**Fig. 2:**
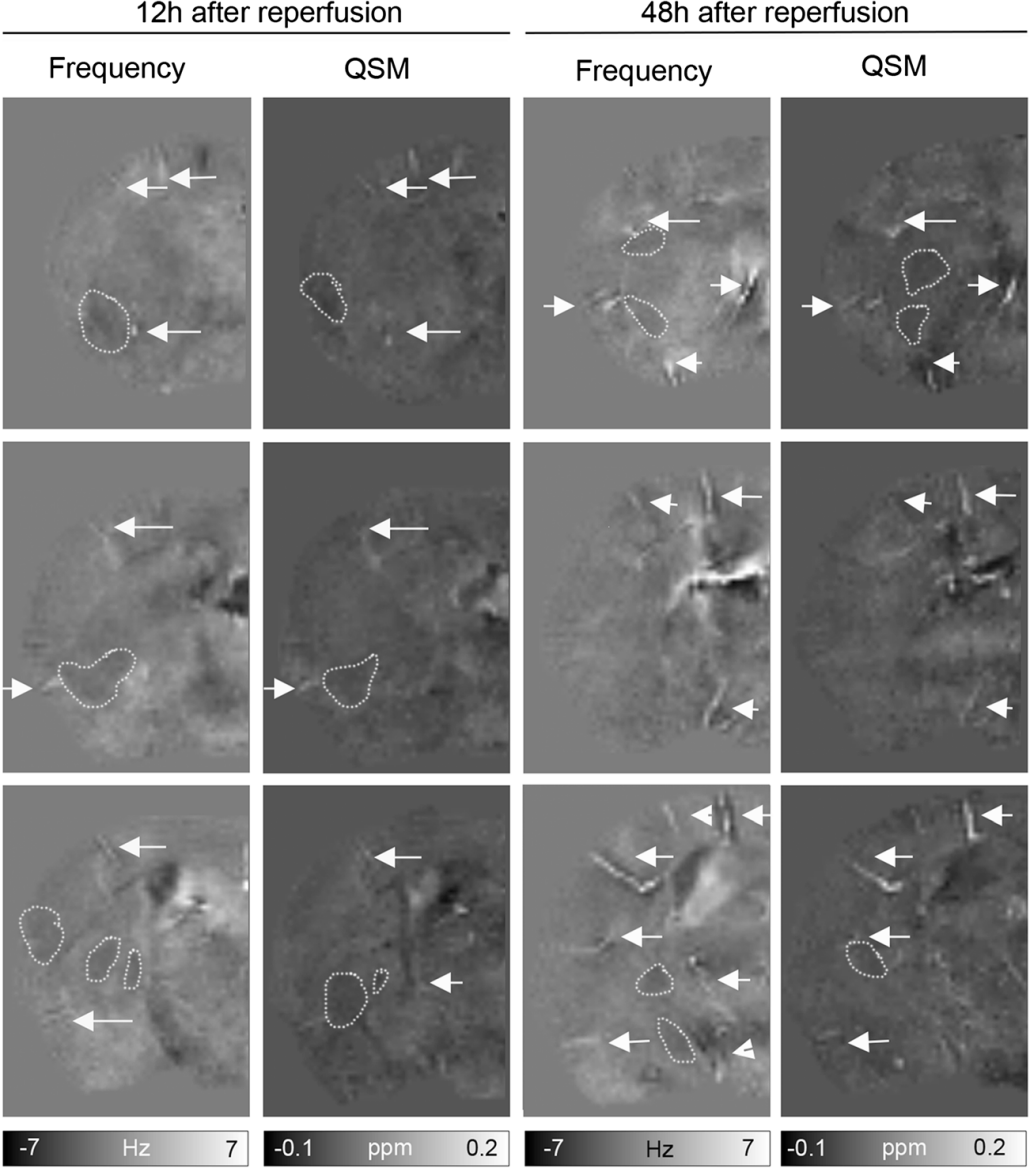
Display of three cross-sections of frequency and quantitative susceptibility maps (QSM) of the ischemic hemisphere of a tMCAO mouse after 12 and 48 h of reperfusion. Prominent vessels with increased magnetic susceptibility (white arrows) occurred more frequently compared to shorter time intervals of reperfusion. Lesions were discernable on both frequency and susceptibility maps (white dotted line) which increased in size

One mouse was excluded from the analysis because no lesion was visible on ADC maps. GRE data of the brain were inspected for the different time periods after reperfusion. Prominent vessels on background-corrected frequency maps and magnetic susceptibility maps of the ischemic hemisphere revealed high frequency and magnetic susceptibility values (Figs. 1 and 2, white arrows). On the frequency maps, they often appeared as white structures surrounded by a dark rim and were mainly found ipsilateral, in the territory supplied by the middle cerebral artery (MCA). On the contralateral hemisphere, vessel-like structures were occasionally observed, but were only faintly visible against tissue background (for example, Fig. 4, 6 h after reperfusion). Moreover, ipsilateral prominent vessels appeared larger in diameter than comparable vessels on the contralateral side. Furthermore, an increased number of prominent vessels were found in the ischemic hemisphere of mice imaged at 12, 24, and 48 h after reperfusion compared to mice imaged at 2, 4, and 6 h after reperfusion.

**Fig. 3:**
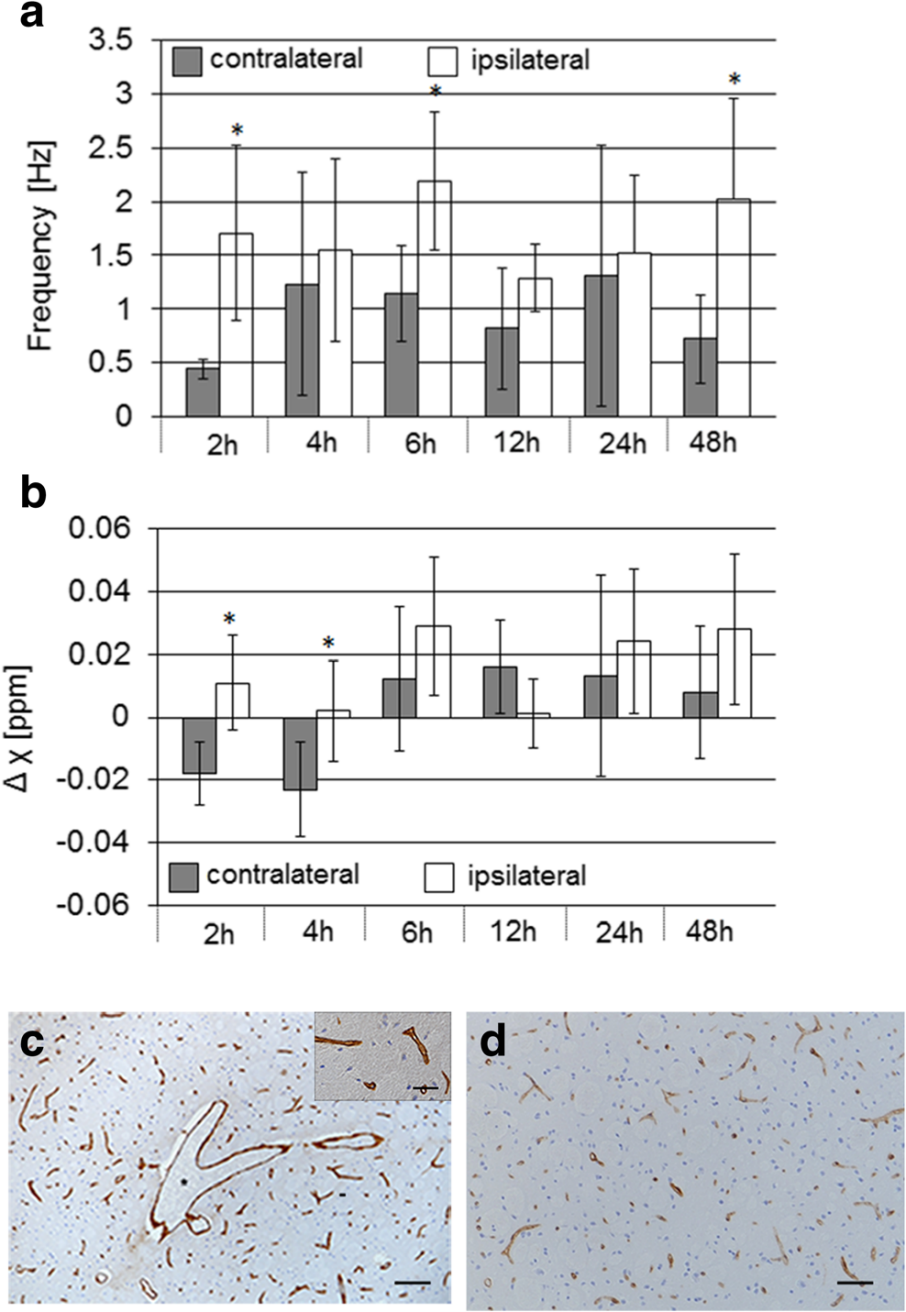
Quantitative analysis of MRI data and assessment of vessels with immunohistochemistry. **a**, **b** VOI analysis of MR frequency values and magnetic susceptibilities in prominent vessels in the ischemic ipsilateral and contralateral hemisphere at different reperfusion intervals, respectively. Bar graphs represent mean ± SD. *p < 0.05 compared to contralateral side. **c**, **d** Representative anti-collagen IV (basal membrane) immunohistochemistry of the brain 24 h after reperfusion. **c** Larger vessels are dilated (asterisk) and endothelial cells in capillaries are swollen and the vessel lumen are narrowed (insert) on the ipsilateral, **d** while this is not observed at the contralateral side. Bar = 100 μm. Insert bar = 20 μm

VOI analysis revealed significantly higher frequency values at 2, 4, and 48 h after reperfusion as well as higher differences in magnetic susceptibility (relative to CSF) at 2 and 4 h after reperfusion in ipsilateral vessels compared to vessels in the contralateral hemisphere (Fig. 3a, b).

Examination of brain sections after collagen IV staining demonstrated dilation of larger vessels on the ipsilateral compared to the contralateral side (Fig. 3c). In addition, capillaries showed swollen endothelial cells and narrowed vessel lumen (Fig. 3c, zoom in) compared to contralateral capillaries in equivalent locations, for which vascular lumen was not affected (Fig. 3c, d).

### Detection of Tissue Changes on Frequency and Susceptibility Maps

Inspection of the background-field corrected frequency maps revealed ipsilateral tissue areas of decreased frequency values at all investigated time points (Figs. 1 and 2, dotted line). The contrast changes were more apparent at 24 and 48 h after reperfusion compared to earlier time points. Similarly, in QSM areas of low magnetic susceptibility, values were observed at 24 and 48 h after reperfusion, while at earlier time points, such areas were only occasionally visible (Figs. 1 and 2). Areas of contrast change confined within the MCA territory; however, the location and extent largely varied within the groups of animals investigated at an individual time point as well as between the different time points.

**Fig. 4:**
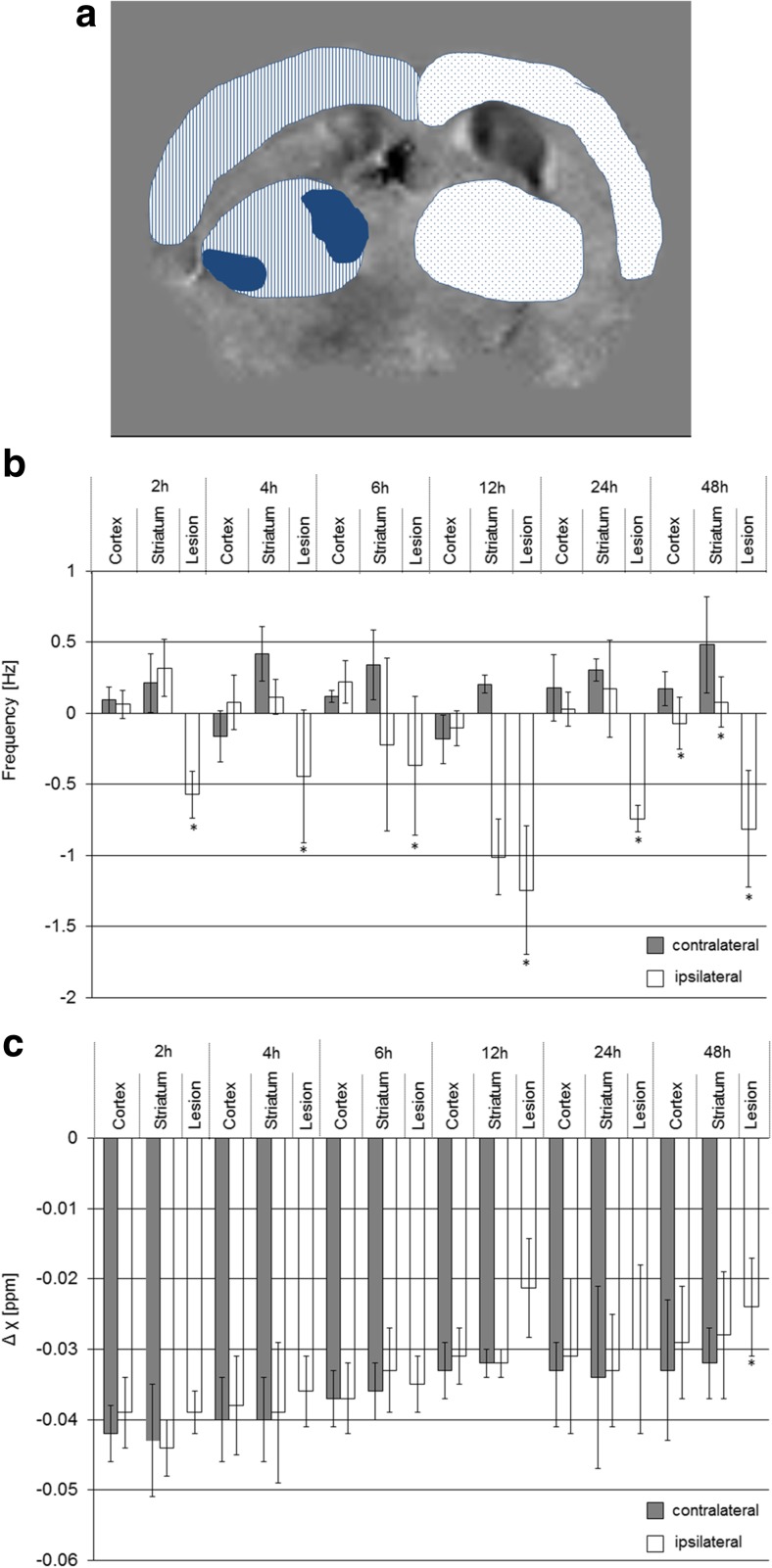
Quantitative analysis of MRI data of different brain regions. **a** Exemplary VOIs selected on the frequency maps. Cortex and striatum were identified on the ischemic hemisphere (*striped pattern*) and the contralateral hemisphere (*dotted pattern*), while excluding areas of markedly reduced frequencies (*blue areas*). VOI analysis of **b** MR frequency values and **c** differences in magnetic susceptibilities (to CSF) in different brain regions at different reperfusion intervals. Bar graphs represent mean ± SD. *p < 0.05 compared to contralateral side

Quantitative analysis demonstrated significantly different frequency values between the lesion areas of regional tissue changes and the contralateral striatum at all time points, while for differences in magnetic susceptibility, a statistical difference was seen at 48 h after reperfusion only (Fig. 4). Values of the cortex and striatum in the ipsilateral hemisphere (excluding the area of regional tissue change) were not statistically different from the values of the corresponding areas on the contralateral side for both contrasts.

### Extent of Regional Contrast Changes for Different MRI Contrasts

**Fig. 5:**
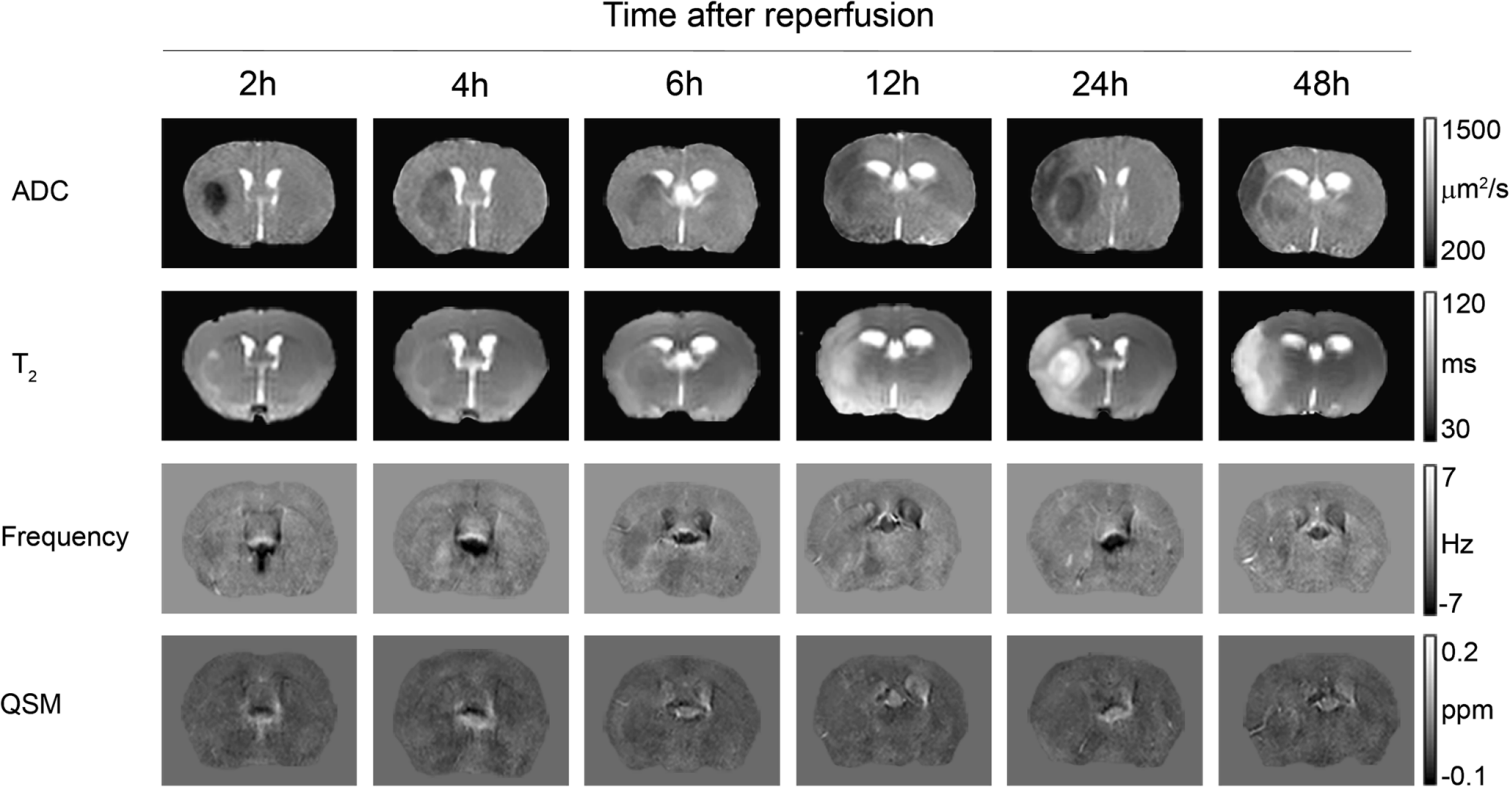
Contrast changes observed on parametric maps of ADC, *T*_2_ relaxation time constant, background-field corrected MR frequency, and magnetic susceptibility (QSM) following 1 h of tMCAO and different intervals of reperfusion. The ischemic lesion first appears on the ADC map as an area with significantly reduced ADC at 2 h after MCAO, before it becomes apparent by increased *T*_2_ values on the *T*_2_ map at 6 h after MCAO. Regional but comparatively smaller contrast changes are discernable on MR frequency and quantitative susceptibility maps from 2 h after reperfusion onwards

**Fig. 6:**
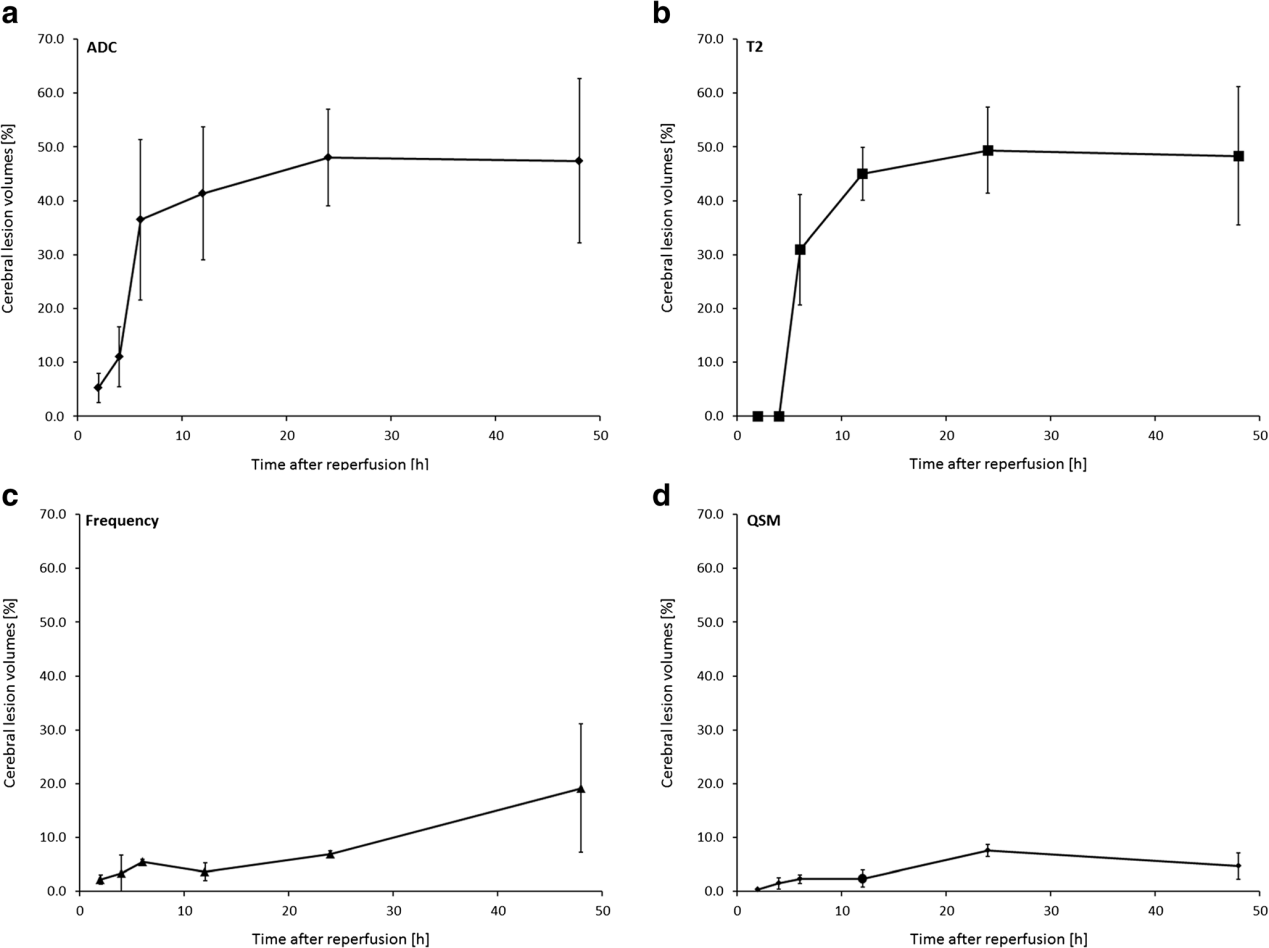
Temporal evolution of the different MRI contrasts after 1 h of tMCAO and different periods of reperfusion. Percentage changes of the extent of regional contrast change (edema corrected) from a region-of-interest analysis of maps of **a** ADC, **b** *T*_2_ relaxation time constant, **c** background-field corrected MR frequency, and **d** magnetic susceptibility (QSM). Displayed are mean ± standard deviations

We measured the extent of contrast changes on frequency and quantitative susceptibility maps and compared it to the cerebral lesion volumes obtained on ADC and *T*_2_ maps (Figs. 5 and 6). Among all MRI contrasts, the ischemic lesion became first visible on ADC maps as areas of decreased ADC at 2 h after reperfusion followed by a steady growth of the lesion until 24 h after reperfusion (Figs. 5 and 6a). On *T*_2_ maps, a lesion with increased *T*_2_ values became the earliest discernable at 6 h after reperfusion with growth until 24 h after reperfusion (Figs. 5 and 6b). The final cerebral hemispheric lesion volumes determined on ADC maps at 48 h after reperfusion were not significantly different from the volumes obtained from *T*_2_ maps (mean ± SD, 47.4 ± 15.2% ADC vs. 48.3 ± 12.8% *T*_2_; *p* = 0.914). In contrast, regions of contrast change as seen on frequency and quantitative susceptibility maps varied considerably in extent and temporal trajectory. Areas of decreased frequency values and magnetic susceptibility were first discernable at 2 h after reperfusion (Figs. 5 and 6c, d). Growth of these regions was only minor on frequency and quantitative susceptibility maps until 48 h after reperfusion, and the extent was at all time points lower compared to the lesion volumes seen on ADC and *T*_2_ maps. The final extent after 48 h after reperfusion was significantly lower on quantitative susceptibility compared to frequency maps (19.1 ± 12.0% frequency vs. 4.7 ± 2.4% QSM; *p* = 0.003).

## Discussion

In the current study, high-resolution MR frequency and quantitative susceptibility maps of the mouse brain were generated after 1 h of tMCAO and different time points after reperfusion. On both maps, prominent vessels were seen with increased frequencies and magnetic susceptibilities. In addition, we observed evolutions of regional contrast, which differed in appearance from those seen on ADC and *T*_2_ relaxation time constant maps.

### Increased Susceptibility of Prominent Cerebral Vessels

Appearance of prominent vessels in the brain of patients with ischemic stroke on SW images and quantitative susceptibility maps have been described recently [17–19]. Their occurrence has been attributed to an increase in the oxygen extraction fraction (OEF) and correlation to misery perfusion indicated by PWI [18]. The increased oxygen extraction leads to higher deoxyhemoglobin concentrations in veins, which increase magnetic susceptibility locally [15]. It has therefore been suggested that these prominent vessels demarcate the penumbra that can be salvaged by vessel recanalization and that SWI can be used to predict infarct growth [17, 19].

In the current study, we also demonstrated the occurrence of prominent vessels in the mouse brain following transient ischemia and reperfusion. Vessels were better visible, appeared wider, and had significantly higher MR frequency values and larger differences of magnetic susceptibility relative to CSF on the ipsilateral side compared to vessels in the contralateral unaffected hemisphere (Figs. 1, 2, and 3). Prominent vessels were mainly observed in the surroundings of the core of the lesion. Reperfusion is associated in part by incomplete blood flow of the microvasculature (no-reflow phenomenon). Examination of brain section stained for the basal membrane demonstrated vasodilation of larger vessels in the ischemic ipsilateral hemisphere, particularly at 24 and 48 h after reperfusion, together with swollen capillaries with narrowed lumen. These findings are in agreement with previous studies which described capillary constriction and impaired capillary reflow as a consequence of pericyte contraction [40, 41]. Thus, our observation of prominent vessels around the lesion after restoration of cerebral blood flow is compatible of lower tissue oxygen availability due to compressed capillaries in the ischemic tissue, which is compensated by an increased oxygen extraction in the surrounding area. As an incomplete blood supply is contributing to the propagation of the ischemic lesion, treatments are required that ameliorate pericyte constrictions and capillary compression to fully restore tissue function after reperfusion. The role of prominent vessels after reperfusion need to be further investigated. Thus, prominent vessels are an important indicator of underlying microvascular pathology and QSM and frequency maps might be used as a proxy of underlying microvascular pathology.

### Detection of Tissue Changes on Frequency and Susceptibility Maps

We also observed decreased frequency values and smaller susceptibility differences relative to CSF in brain tissue regions, which has so far not been reported in patients with ischemic stroke. One reason for this could be that we performed our study at a field strength of 4.7 T, which is higher than those commonly used in clinical studies (usually 1.5 T). As the effects of magnetic susceptibility are proportional to the applied magnetic field, we are thus more sensitive regarding detectability of differences in magnetic susceptibility between tissues. Furthermore, we used a higher spatial resolution (100 μm) compared to clinical studies (usually > 1 mm), which again helps to delineate small morphological features. The accompanying loss in signal-to-noise ratio was compensated for by using higher field strength and a cryogenic transmitter-receive RF coil [32]. However, also differences in principle function between our animal model and typical clinical scenarios, including patient selection and stroke etiology, type and location may be responsible for the lack of this observation in patients.

The appearance of regions with decreased frequency values and smaller susceptibility differences was more apparent at 24 and 48 h after reperfusion compared to the earlier investigated time points, but was highly variable between individual animals. Moreover, the growth rate and extent of these areas were significantly smaller on frequency and quantitative susceptibility maps compared to ADC and *T*_2_ maps. The underpinnings of these changes on susceptibility and frequency maps are currently unclear. Since cerebral ischemia is followed by microstructural changes at different stages with edema formation, cell death, and tissue destruction and phagocytosis of debris resulting in cavitation with surrounding gliosis [42–44], this complex chain of processes is difficult to capture with single frequency or susceptibility values. On the other hand, compartmental water shifts due to cytotoxic and vasogenic edema can be detected by DWI and *T*_2_ mapping, respectively [6, 7]. Interestingly, we did not observe a spatial congruence between regions of increased *T*_2_ and reduced ADC values with areas of reduced MR frequency values and differences in magnetic susceptibility. Thus, both contrasts do likely not represent the ischemic lesion as distinct to *T*_2_-weighted imaging and ADC, where a good correlation to the histopathological lesion has been demonstrated [45]. It might be speculated that in the regions, which are at the core of the lesion, oxygen is not extracted [46], which increases oxygenated hemoglobin concentrations in those areas and produces a diamagnetic shift of susceptibility [47]. Clearly, the relevance of these speculations and the associated cellular underpinnings need to be investigated in further studies.

Taken together, we were able in our study to identify characteristic changes of magnetic susceptibility in the mouse brain after transient ischemia followed by reperfusion. Since QSM can be reconstructed from GRE data, there is no extra penalty in acquisition time to reconstruct such maps. In addition, several approaches for accelerating GRE acquisitions have been proposed for human imaging [48–51]. Currently, clinical application of QSM is hampered due to its numerical complexity and computational cost associated with the post-processing procedure. More recently, novel algorithms have been proposed, which allow rapid online reconstruction of susceptibility maps directly after data acquisition, enabling instant evaluation by medical personal [52]. Thus, QSM appears promising as a useful post-processing tool to evaluate GRE data for the diagnosis and follow-up of patients with ischemic stroke.
